# Silencing XIAP suppresses osteosarcoma cell growth, and enhances the sensitivity of osteosarcoma cells to doxorubicin and cisplatin

**DOI:** 10.3892/or.2021.8213

**Published:** 2021-10-29

**Authors:** Yang Qu, Peng Xia, Shanyong Zhang, Su Pan, Jianwu Zhao

Oncol Rep 33: 1177-1184, 2015; DOI: 10.3892/or.2014.3698

Following the publication of this paper, it was drawn to the Editors attention by a concerned reader that the western blotting data featured in Figs. 2B and 4D, the flow cytometric data in Fig. 4A and the tumour images shown in Fig. 6A were strikingly similar to data appearing in different form in other articles at different research institutes. Owing to the fact that the contentious data in the above article were already under consideration for publication, or had already been published, elsewhere prior to its submission to *Oncology Reports*, the Editor has decided that this paper should be retracted from the Journal. After having been in contact with the authors, they agreed with the decision to retract the paper. The Editor apologizes to the readership for any inconvenience caused.

